# Construction of lncRNA prognostic model related to cuproptosis in esophageal carcinoma

**DOI:** 10.3389/fgene.2023.1120827

**Published:** 2023-04-13

**Authors:** Liming Zhang, Ling Zong, Wenhui Li, Lu Ning, Yajun Zhao, Shaoqiang Wang, Lina Wang

**Affiliations:** ^1^ Department of Clinical Medicine, Jining Medical University, Jining, China; ^2^ Department of Thoracic Surgery, Affiliated Hospital of Jining Medical University, Jining Medical University, Jining, China; ^3^ Medical Research Center, Affiliated Hospital of Jining Medical University, Jining Medical University, Jining, China

**Keywords:** cuproptosis-related lncRNAs (CRLs), esophageal carcinoma (ESCA), prognosis, prognostic model, bioinformatics analysis

## Abstract

**Background:** Esophageal carcinoma (ESCA) is one of the most prevalent malignant tumors in the world. The prognosis of patients has significantly improved with the development of surgery, targeted therapy and immunotherapy. But the 5-year survival rate of ESCA patients is still incredibly low. Cuproptosis is a type of mitochondrial cell death induced by copper. It is unclear how cuproptosis-related lncRNAs (CRLs) affect ESCA prognosis.

**Methods:** In this study, we obtained the clinical data of ESCA patients, the transcriptome data from TCGA and identified CRLs by co-expression analysis, lasso regression, and cox regression analysis, to build a prognostic model. Then we validated the prognostic model using the Kaplan-Meier curve, cox regression analysis, and ROC, to create a nomogram based on risk score to forecast the prognosis of ESCA. Next, the immune escape of the CRLs was examined using the TIDE algorithm to assess its sensitivity to possible ESCA medications.

**Results:** To predict the prognosis of ESCA patients, we created a predictive model using 6 CRLs (AC034199.1, AC125437.1, AC107032.2, CTBP1-DT, AL024508.1, and AC008610.1), validated by the Kaplan-Meier and ROC curves. The model has a higher diagnostic value compared to other clinical features. The 6 CRLs expressed high in TCGA and ESCA specimens. Enrichment analysis revealed CRLs largely contributed to the interaction between cytokines and their receptors as well as complement coagulation cascades. The immunity escape analysis demonstrated that immunotherapy had a worse effect in the low-risk group than in the high-risk group. Additionally, we screened out potential antineoplastic drugs according to the results of the immunoassay and obtained 5 drugs, including CP-466722, crizotinib, MS-275, KIN001-135, and CP-466722.

**Conclusion:** The prognosis of patients with ESCA can be correctly predicted by the 6 CRLs chosen from this investigation. It lays the groundwork for more investigation into the ESCA mechanism and the identification of novel therapeutic targets.

## Introduction

One of the most prevalent malignant tumors worldwide is ESCA. The incidence of esophageal cancer and overall mortality are currently ranked sixth and seventh, respectively, in the world (Sung et al., 2021). Esophageal squamous cell carcinoma (ESCC) and esophageal adenocarcinoma (EAC) are the two subtypes of esophageal cancer according to the pathological classification (Arnold et al., 2015). Radical esophagectomy is currently the primary treatment for non-metastatic esophageal cancer; while radiotherapy and chemotherapy are the primary strategies for advanced esophageal cancer. However, the outcomes are unfavorable (Cooper et al., 1999; Sudo et al., 2014). Therefore, investigating the ESCA early screening markers and treatment targets is very important.

Cell death, which can be classified as apoptosis, necrosis, autophagy, and ferroptosis, is crucial for fundamental physiological processes like development, immunity, and tissue homeostasis (Stockwell, 2019; Kist and Vucic, 2021). Cuproptosis, a copper-triggered mode of mitochondrial cell death, was first identified by Tsvetkov et al. (2022) on March 17, 2022, which differs from the types of cell death previously described. A ubiquitous metal element with redox activity, copper contributes to a number of biological processes by either giving or taking electrons (Li and Trush, 1993). The production of iron-sulfur cluster proteins is downregulated as a result of copper direct binding to the fatty parts of the tricarboxylic acid (TCA) cycle, which causes the accumulation of fatty acylated proteins, produces protein toxic stress, and ultimately results in cell death. In addition, copper is a vital mineral nutrient for all life. Compared to healthy cells, cancer cells require more copper (Pavithra et al., 2015; Huang et al., 2019; Atakul et al., 2020; Feng et al., 2020). According to the research, a copper shortage prevents tumor growth and angiogenesis (Yu et al., 2019). It is evident that cuproptosis is crucial for the emergence and growth of malignancies.

About 98% of the human transcriptional genome is made up of non-coding RNAs (ncRNAs), which play important roles in numerous physiological and pathological processes, especially in cancer. As a result, it has emerged as one of the primary areas of contemporary research (Slaby et al., 2017). Long non-coding RNAs (LncRNAs) are ncRNAs with a length of more than 200 bp and crucial for both transcriptional activation and silencing. LncRNAs have been implicated in a variety of diseases, including cancer (Barik et al., 2021). LncRNAs are also involved in tumor medication resistance (Hosseini et al., 2021). However, there is fairly few research focusing on the connection between CRLs and the prognosis of ESCA patients.

In this study, we downloaded and searched TCGA database for CRLs, built a prognostic model, and tested its predictive power. The probable mechanism of CRLs in ESCA was further investigated by enrichment analysis and immune escape analysis.

## Materials and methods

### Data collection and identification of the CRLs

Download the clinical data of 183 patients, including age, sex, grade, stage, tumor lymph node metastasis, survival time, survival status, and more. Download the ESCA HTSeq-Count data from TCGA database (https://portal.gdc.cancer.gov/) (Tomczak et al., 2015), which includes 161 ESCA tissues and 11 normal tissues. From earlier literature, the copper death-related genes (NFE2L2, NLRP3, ATP7B, ATP7A, SLC31A1, FDX1, LIAS, LIPT1, LIPT2, DLD, DLAT, PDHA1, PDHB, MTF1, GLS, CDKN2A, DBT, GCSH, DLST) were retrieved (Aubert et al., 2020; Dong et al., 2021; Ge et al., 2022; Tsvetkov et al., 2022). It is possible to get the lncRNA comment file from the GENCODE website (https://www.gencodegenes.org/). CRLs were identified using the R language (4.1.3) limma package, with correlation coefficient >0.4 and *p* < 0.001 serving as the screening criteria (Ritchie et al., 2015). The co-expression data was then shown by the ggplot2 and ggalluvial package.

### Construction of prediction features of CRLs

We divided TCGA ESCA data into training and testing groups at random. Then constructed a prognostic model using training group data while validating with the testing group and entire group data. In order to find lncRNAs associated with the prognosis of ESCA (*p* < 0.05), we first utilized univariate cox analysis on training group data, then visualized the results to create a forest map. The resulting lncRNAs are subjected to Lasso cox regression analysis, cross-validation, and risk model establishment. The target lncRNAs were chosen by cox regression analysis, and the risk score of ESCA patients is determined in accordance with the prognosis model. Risk score = (Expi × bi). (Exp: expression level of model gene; bi: model gene coefficient). Finally, the results are displayed by the ggplot2 package.

### Validation of risk models

Patients in training groups and testing groups were split into a high-risk group and a low-risk group based on their median risk score. The difference in overall survival (OS) and progression-free survival (PFS) between the 2 groups of ESCA patients was analyzed by the Kaplan-Meier curve. The predictive value of the risk prediction signature was assessed *via* univariate and multivariate cox regression analysis. Next, the survival status map and lncRNA expression heat map based on risk score were created using the pheatmap package. To assess the diagnostic utility of cuproptosis-associated lncRNAs, the receiver operating characteristic curve (ROC) and area under the curve (AUC) were obtained by the SurvivalROC package.

### Construction and calibration of the nomogram

Using the rms package, we combine the risk score with the clinicopathological traits of ESCA patients to create a nomogram. Next, draw the calibration curve for 1, 3, and 5-year to assess the discrepancy between the predicted and actual results. Finally, create the C-Index curve to confirm the nomogram model’s predictive power.

### GO and KEGG analysis

We conducted the GO and KEGG enrichment analysis of the prior lncRNAs using the clusterProfiler package, enrichplot, and other tools. We investigated the biological process (BP), cellular component (CC), molecular function (MF), and KEGG pathways.

### Immune-related function analysis, tumor immune dysfunction and exclusion (TIDE) and potential drug screening

We evaluated immune-related functions and presented the findings using the limma, GSVA, and GSEABase packages. To download the TIDE website’s tumor immune dysfunction and rejection files, utilize the limma and ggpubr packages to explore the possibility of training and testing different immune escapes. To determine which medications were different between the training and testing groups, we used the limma package, ggpubr package, and pRRophetic package to screen possible pharmaceuticals.

### Human esophageal cancer specimen collection

We collected tumor tissue and paracancerous tissue samples from 40 patients with esophageal carcinoma who underwent surgery in our hospital. The identical patients’ paraneoplastic non-tumor tissues were taken simultaneously. No preoperative treatments were given to the patients. All the patients signed informed consent forms. The Affiliated Hospital of Jining Medical University’s Ethics Committee evaluated and approved this work (Approval number: 2021-11-C009).

### RNA extraction and qRT-PCR analysis

TRIzol reagent was used to extract total cellular RNA (Invitrogen, United States). A commercial cDNA synthesis Kit (SuperScript First-Strand Synthesis System, Thermo Fisher Scientific) was utilized to generate cDNAs through reverse transcription. The SYBR Green Assay Kit was used to detect the presence of mRNA (TAKARA, Japan). Using the Biosystems ViiA7 Sequence Detection System, the qRT-PCR procedure was carried out 3 times. Relevant primer synthesis are as follows:

AC034199.1 forward primer: 5′-TAG​AGC​CCT​CGC​ACC​TCT​TA-3′, AC034199.1reverse primer:5′-TGTACCAGGGGTGTTTCCCT-3’;

AC125437.1 forward primer: 5′-GCT​CCT​TCA​TGT​CCA​GCC​ATA-3′, AC125437.1reverse primer:5′-GGCTACGTGGCATTAGGAGT-3’;

AC107032.2 forward primer: 5′-TTT​TGG​ATT​CTG​AAG​ATT​CAG​TGC-3′, AC107032.2reverse primer:5′-GGAAAGACAAACAGCCACATT-3’;

CTBP1-DT forward primer: 5′-TCT​AAG​ATC​GGG​CTG​CCG​AG-3′, CTBP1-DT reverse primer:5′-GTTCCCTCCTTCATGACTCCC-3’; AL024508.1 forward primer: 5′-GCC​ACA​GAC​CAA​GAA​CCC​AT-3′, AL024508.1 reverse primer:5′-CTACACCCGAGGAAAGCACT-3’;

GAPDH forward primer:5′-CCAGCAAGAGCACAAGAGGAA-3’

GAPDH reverse primer:5′-ATGGTACATGACAAGGTGCGG-3’.

## Results

### Identification of CRLs and construction of a prognostic signature

**FIGURE 1:**
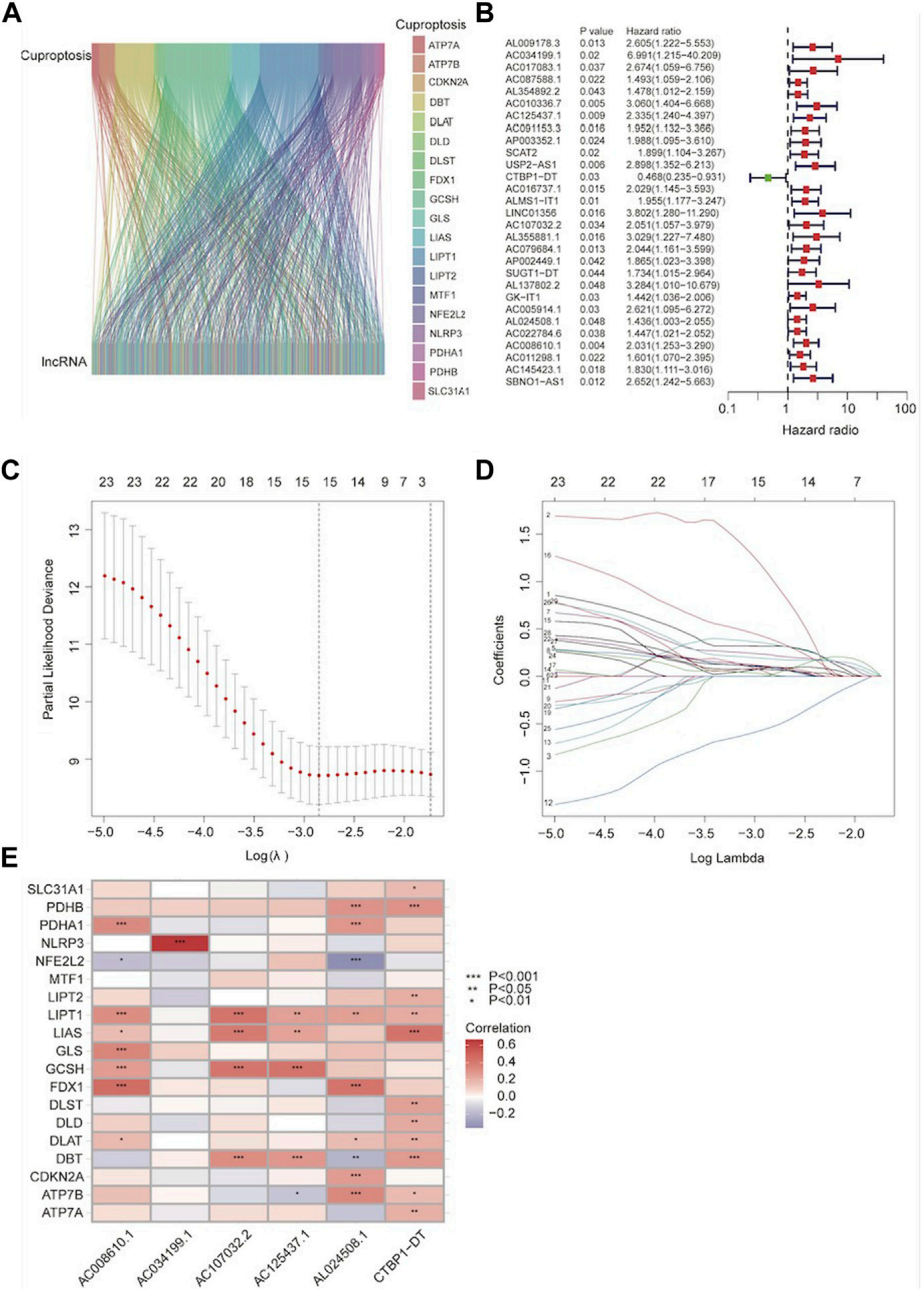
Identification of the cuproptosis-related lncRNAs (CRLs). **(A)** Co-expression of lncRNA and cuproptosis-related genes. **(B)** The forest map shows the lncRNA of different risk groups; red represents high-risk lncRNAs and green represents low-risk lncRNAs. **(C)** A general cross-validation curve of the paired likelihood deviance. **(D)** LASSO coefficients of prognostic genes. **(E)** A correlation heatmap revealed the association between CRLs and cuproptosis-related genes. Positive correlations are shown in red while negative correlations are represented in blue.

16773 lncRNAs and their expression data were obtained after downloading the TCGA ESCA expression data file and the lncRNA annotation file from the GENCODE website. We performed co-expression analysis by the screening thresholds of Pearson R > 0.4 and *p* < 0.001 to identify 553 lncRNAs that co-expressed with the cuproptosis genes (Supplementary Table S1). The co-expression relationship was shown by the Sankey map (Figure 1A). We conducted a univariate cox analysis on the training group data after randomly dividing the ESCA data into training and testing groups. The following 29 lncRNAs were found to be associated with prognosis: AL009178.3, AC034199.1, AC017083.1, AC087588.1, AL354892.2, AC010336.7, AC125437.1, AC091153.3, AP003352.1, SCAT2, USP2-AS1, CTBP1-DT, AC016737.1, ALMS1-IT1, LINC01356, AC107032.2, AL355 SBNO1-AS1, AL024508.1 b, AC022784.6, AC008610.1, AC011298.1, AC145423.1, AC005914.1, and AC011298.1 (Figure 1B). Using the results from the previous steps, we utilized lasso regression to identify the lncRNAs with the highest predicted value (Figures 1C, D). By drawing a heat map, we were able to see the significant correlation between 6 of these lncRNAs and cuproptosis-related factors (Figure 1E). Finally, lncRNA expression was used to construct the risk score. Risk score = AC034199.1 × 2.53383298403335+AC125437.1 × 0.630085759483287+ CTBP1-DT×0.88312435523092+AC107032.2 × 0.937659185430685+ AL024508.1 × 0.323078458616612+AC008610.1 × 0.677274791962651.

### Verification of prognostic model

**FIGURE 2:**
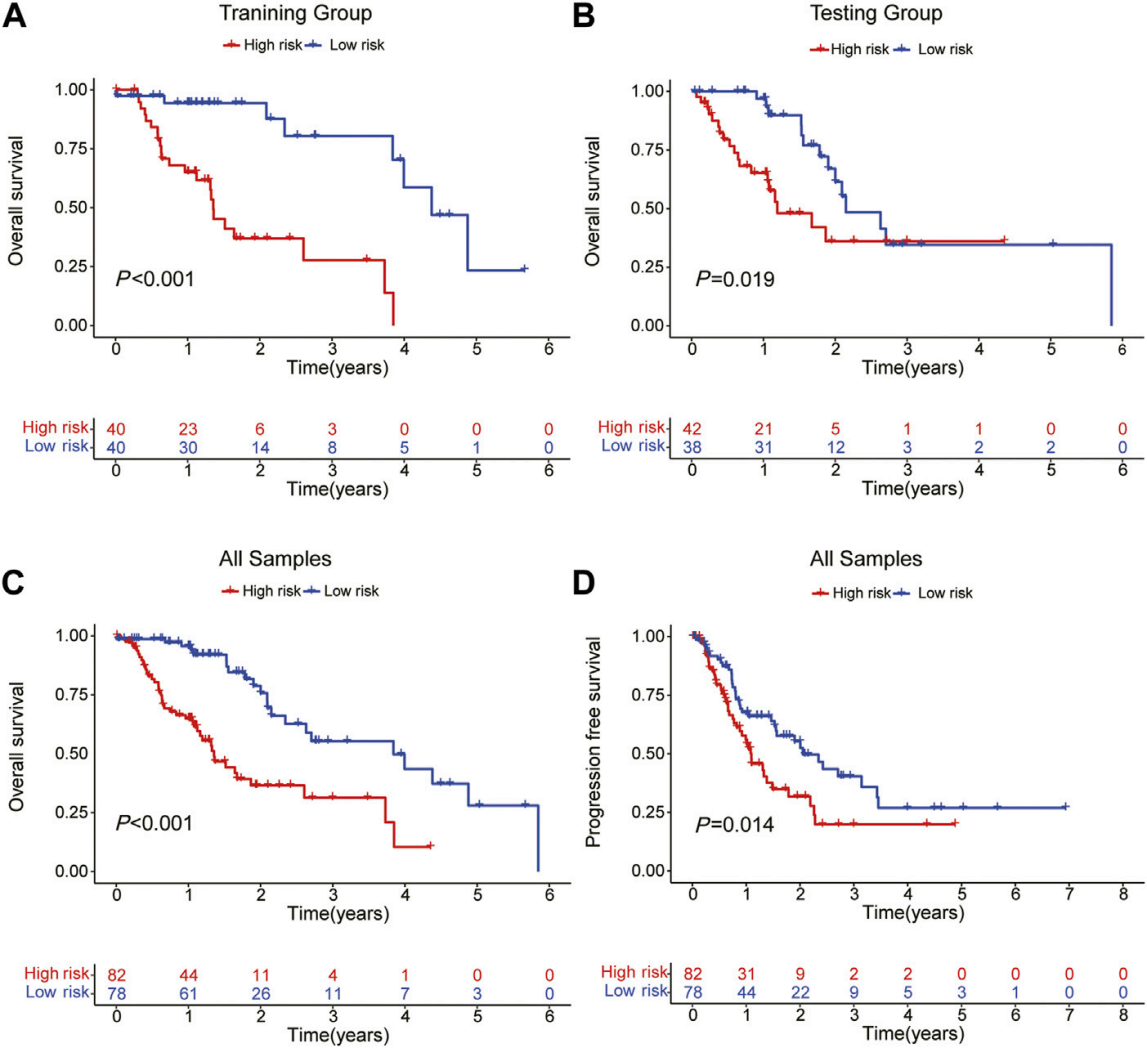
OS and PFS in each subgroup predicted by Kaplan-Meier survival curves. **(A)** OS in the training group. **(B)** OS in the testing group. **(C)** OS in all samples. **(D)** PFS in all samples.

**FIGURE 3:**
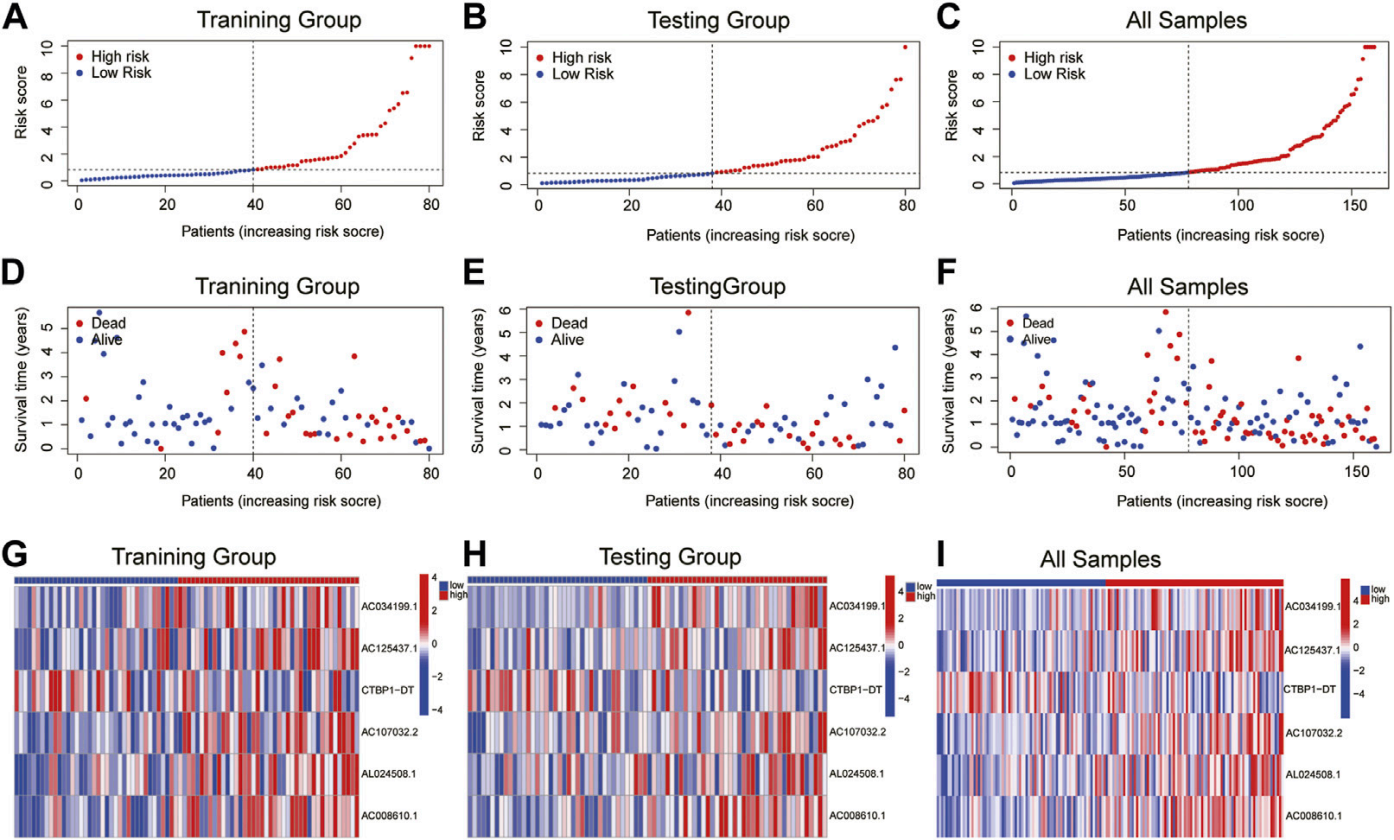
The distribution of OS risk scores and heat maps of 6 lncRNAs. **(A–C)** The distribution of OS risk scores. **(D–F)** Survival time and status. **(G–I)** Heat maps of 6 lncRNA expressions.

The patients were split into high-risk and low-risk groups based on the computed risk score. The Kaplan-Meier curve was used to examine the OS and PFS of the training group, the testing group, and all groups. The findings demonstrated that the OS and PFS of the high-risk group were noticeably inferior to those of the low-risk group (Figure 2). The distribution of risk ratings and the patients’ survival status in each group were then displayed. The survival time of ESCA patients decreased and the number of fatalities rose as risk scores rose (Figures 3A–F). The expression levels of CRLs in the high-risk group and low-risk group are then displayed on the heat map, which shows that CTBP1-DT is a low-risk lncRNA, whereas AC034199.1, AC125437.1, AC107032.2, AL024508.1, and AC008610.1 are high-risk lncRNAs (Figures 3G–I).

### An independent prognostic indicator for ESCA of the CRLs signature

**FIGURE 4:**
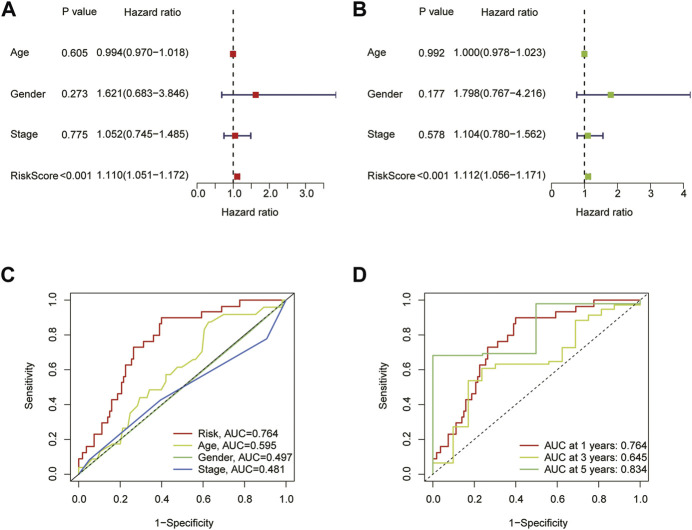
The prognostic value of the signature for ESCA. **(A)** Univariate Cox analysis based on age, gender, stage, and risk scores. **(B)** Multivariate Cox analysis based on age, gender, stage, and risk scores. **(C)** Comparison of the predictive accuracy of risk models and clinicopathological characteristics. **(D)** ROC of the risk characteristic based on a whole-group prediction of 1, 3, and 5-year OS.

To assess the prognostic model’s accuracy in predicting outcomes, we conducted univariate and multivariate cox regression analysis based on age, gender, stage, and risk scores. The results demonstrated that the risk score was a factor that independently affected the outcome of ESCA patients (*p* < 0.001) (Figures 4A, B). Additionally, we employed a ROC curve to assess the diagnostic utility of CRLs in OS. The AUC of risk score was 0.764, which was better than age (0.595), gender (0.497) and staging (0.481) (Figure 4C). And the AUC of 1-year, 3-year, and 5-year were 0.764, 0.645, and 0.834, respectively, showing that the model has very solid diagnostic utility (Figure 4D).

### Construction and validation of a nomogram

**FIGURE 5:**
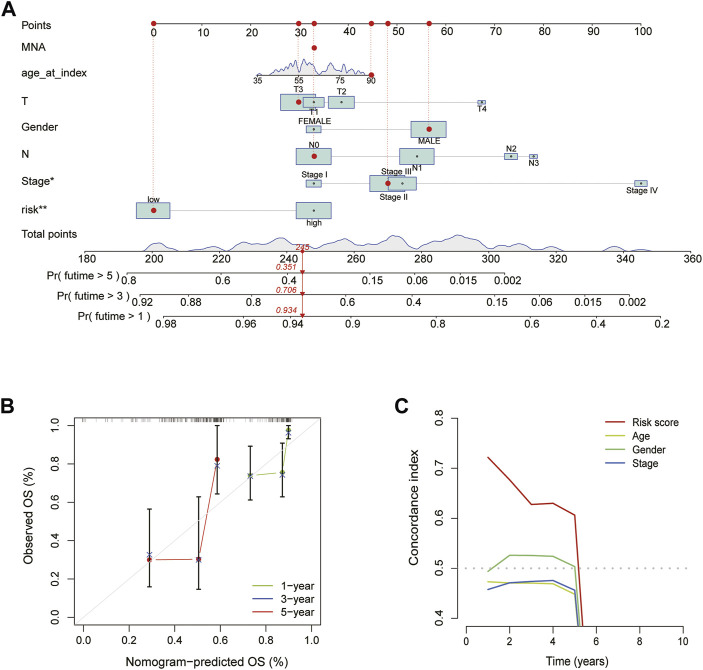
Construction and validation of the nomogram. **(A)** Nomogram predicts the prognosis of patients with ESCA for 1, 3 and 5 years. **(B)** The accuracy of Calibration curves verification results. **(C)** The C-index curve of the risk mode.

We created a nomogram to forecast patients’ OS of 1, 3 and 5-year based on the clinical traits and risk scores of ESCA patients. The graphic reveals that a nomogram may accurately predict patients’ OS at 1, 3 and 5-year (Figures 5A, B). The C-index of risk score was much greater than that of age, gender, stage, and other clinical features, according to the comparison of the indicators performed (Figure 5C).

### Expression of CRLs in TCGA and 40 paired ESCA tissues

**FIGURE 6:**
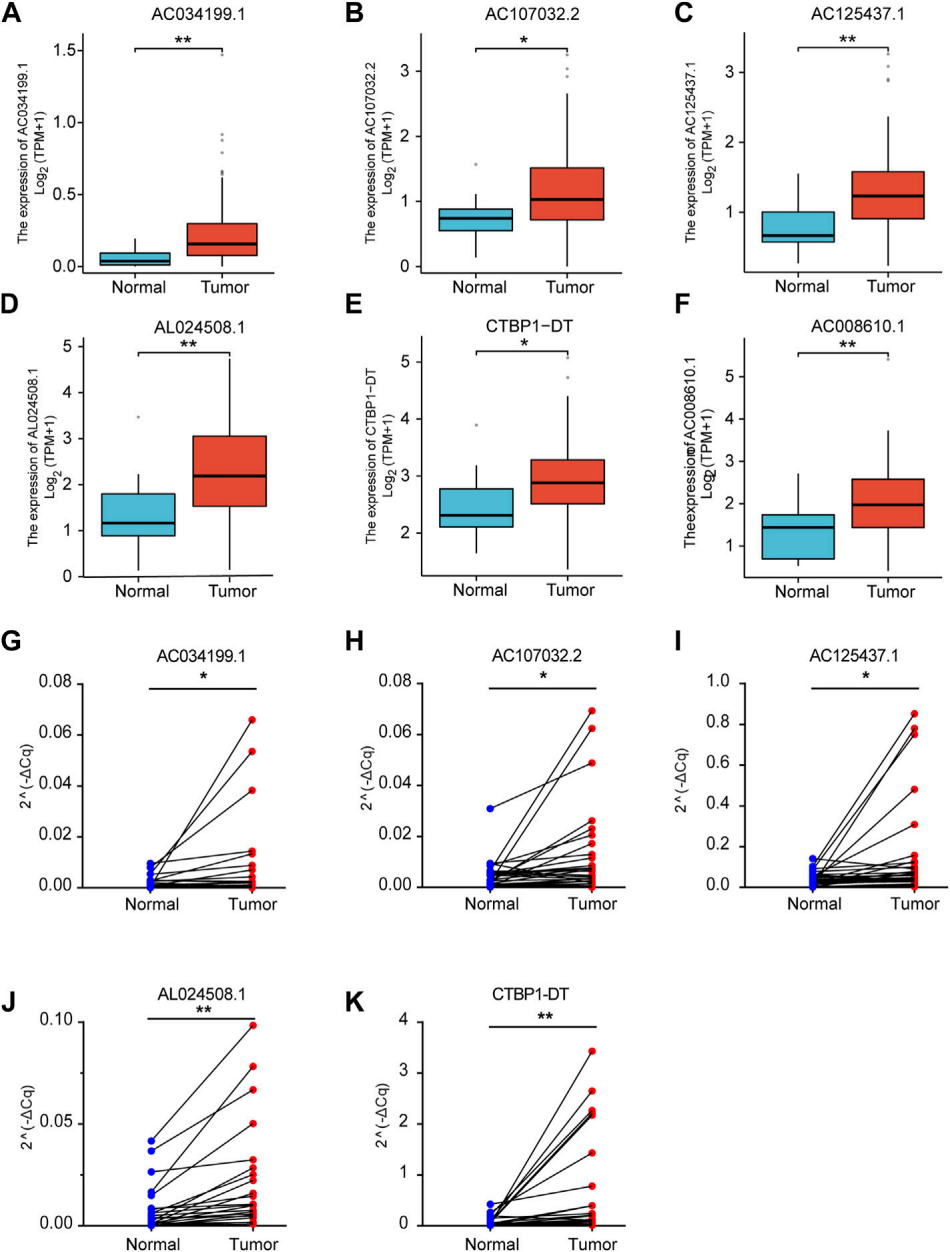
Expression of lncRNAs in TCGA and 40 paired ESCA tissues. The expression of AC034199.1 **(A)**, AC107032.2 **(B)**, AC125437.1 **(C)**, AL024508.1 **(D)**, CTBP1-DT **(E)** and AC008610.1 **(F)** in TCGA. qRT-PCR validation of expression of AC034199.1 **(G)**, AC107032.2 **(H)**, AC125437.1 **(I)**, AL024508.1 **(J)**, and CTBP1-DT **(K)** in 40 paired ESCA tissues.

We analyzed the transcriptome data of TCGA to explore the expression of CRLs in ESCA. The results showed that CRLs were highly expressed in esophageal cancer (Figures 6A–F). To further confirm the expression of the CRLs, the 40 ESCA samples were collected and verified by qRT-PCR. Our validation results showed that the above 6 CRLs were significantly highly expressed in ESCA, which was consistent with the expression in TCGA (Figures 6G–K).

### GO and KEGG analysis

**FIGURE 7:**
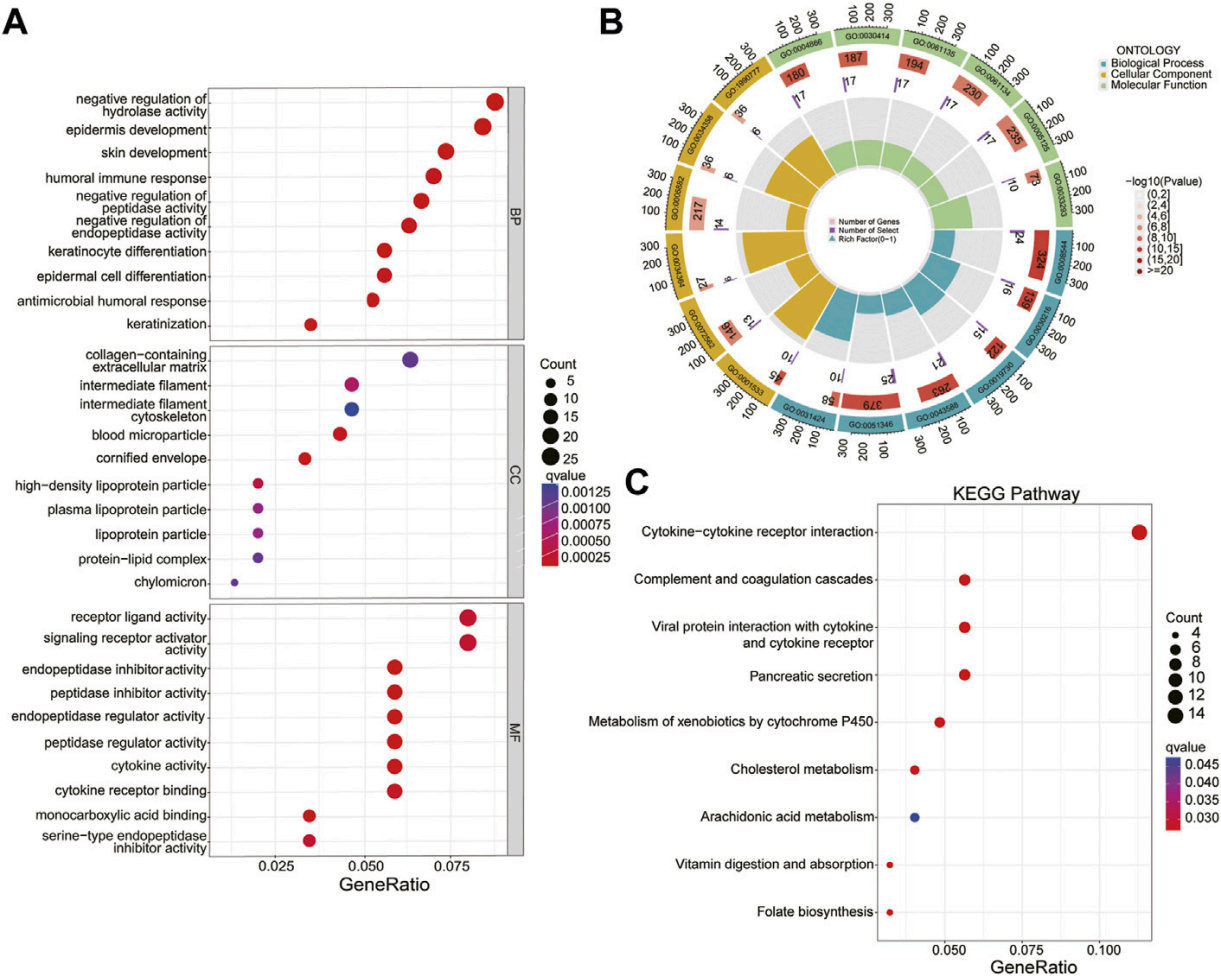
GO and KEGG analysis. **(A, B)** Biological process (BP), cellular component (CC), and molecular function (MF) of CRLs. **(C)** KEGG pathway.

The GO and KEGG analyses showed that in the biological process (BP) category, it was mainly enriched in epidermis development, keratinocyte differentiation and antimicrobial humoral response; In the cellular component (CC) category, mainly enriched in the cornified envelope, blood microparticle and high-density lipoprotein particle; In the molecule function (MF) category, it was endopeptidase inhibitor activity, peptidase inhibitor activity and endopeptidase regulator activity (Figures 7A, B). Genes in the KEGG category were enriched in the cytokine-cytokine receptor interaction complement, coagulation cascades and vitamin digestion and absorption (Figure 7C).

### Immune-related function analysis, TIDE and drug sensitivity analysis

**FIGURE 8:**
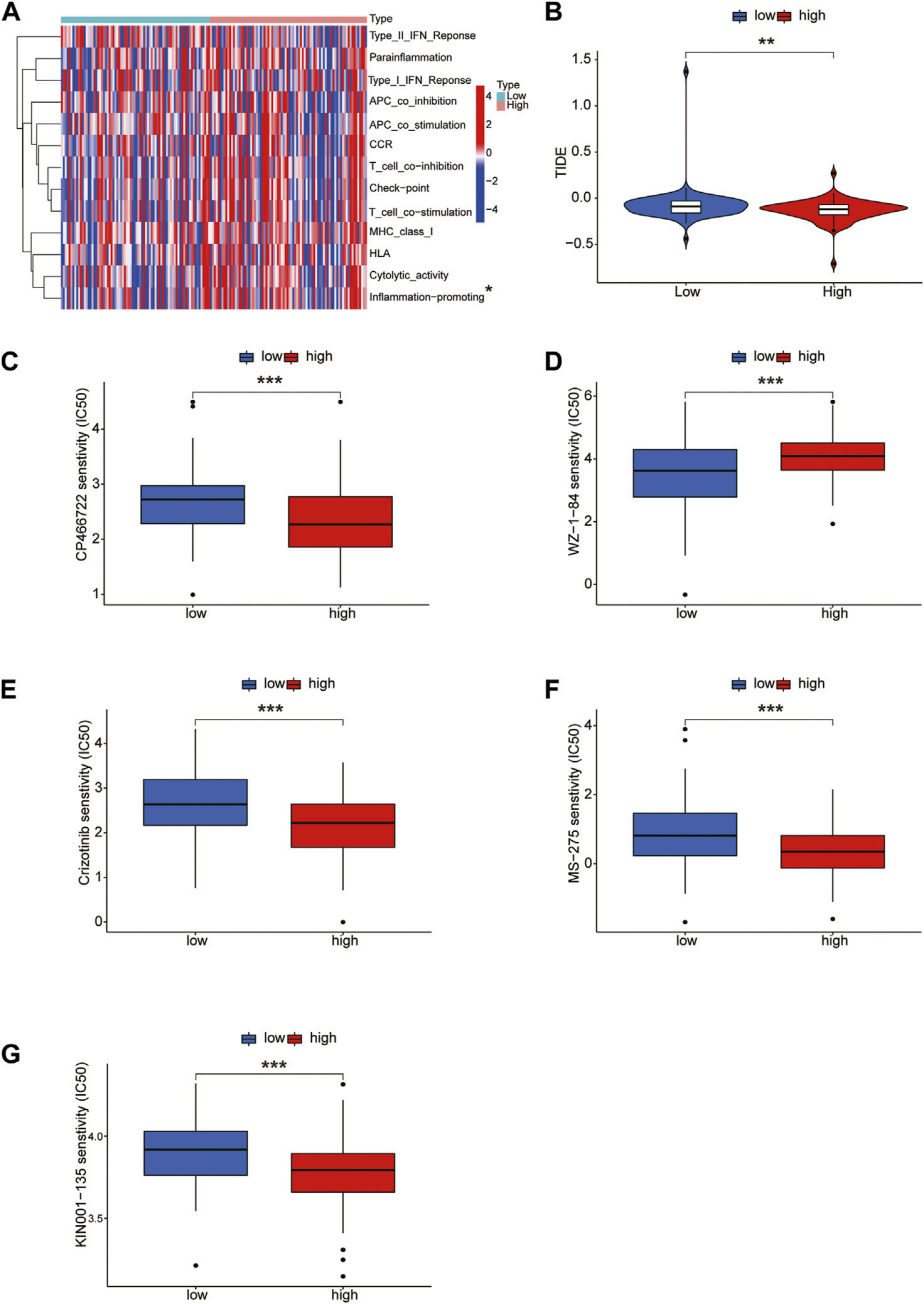
Immune-related function analysis and drug sensitivity. **(A)** Immune-related function analysis of CRLs. **(B)** Tumor immune dysfunction and exclusion (TIDE) algorithm analysis for high-risk and low-risk groups. Drug sensitivity of CP-466722, crizotinib, MS-275, KIN001-135, CP-466722 **(C–G)**.

Next, we conducted an immune-related function analysis, and the results showed that inflammation-promoting had significant differences in risk scores (*p* < 0.05) (Figure 8A). The effectiveness of immunotherapy in the high-risk and low-risk groups was assessed. Accordingly, the low-risk group’s TIDE score was considerably higher than that of the high-risk group, indicating the low-risk group’s immunotherapy effect was less favorable than that of the high-risk group (Figure 8B). In order to identify possible antineoplastic medications, we tested the sensitivity of common anticancer drugs between the 2 groups. The findings revealed that the high-risk group received more benefit from the ATM inhibitor CP-466722, crizotinib, the HDAC inhibitor MS-275, and KIN001-135 whereas the low-risk group received more benefit from the WZ-1-84 (Figures 8C–G).

## Discussion

ESCA is one of the most common malignancies of the digestive tract. Despite advancements in chemotherapy, radiation, and surgery, the 5-year survival rate of ESCA is still under 30% (van Hagen et al., 2012). Therefore, it is crucial to discover fresh biomarkers for ESCA diagnosis. It has been widely acknowledged that copper is a common metal element with redox activity and a vital mineral for all living things. Most of the time, intestinal cells absorb copper from meals. The daily intake of copper in humans is 0.9 mg according to the US Food and Drug Administration. (2016). If the body cells absorb too much Cu^2+^, the production of hydroxyl radicals, the level of reactive oxygen species (ROS) and lipid peroxidation will increase, leading to oxidative stress and apoptosis (Halliwell and Chirico, 1993; Osawa et al., 2021). The body’s copper content maintains a dynamic balance, and when the balance is disrupted, it can produce cytotoxicity and induce cell death through a variety of pathways. As a newly discovered form of cell death, cuproptosis is a copper-triggered modality of mitochondrial cell death. The way that copper causes cell death is through direct binding to the fatty parts of the TCA cycle. This causes an accumulation of fatty acylated proteins and a downregulation of the expression of iron-thio cluster proteins, which in turn causes protein toxic stress and ultimately cell death. LncRNAs are longer than 200 bp and do not code for proteins. Numerous studies have demonstrated that lncRNAs can regulate carcinogenic and tumor inhibitory pathways to influence the occurrence, growth, and spread of cancer (Derrien et al., 2012). However, the function of CRLs in ESCA requires much deeper research.

In this study, we downloaded ESCA data from TCGA database and examined their co-expression, resulting in the identification of 29 lncRNAs associated with cuproptosis. Six CRLs related to the prognosis of ESCA were identified by univariate and multivariate cox regression analysis. DNA damage-upregulated protein (DDUP), a microprotein encoded by the recently discovered LncRNA CTBP1-DT, is crucial for DNA damage repair through PRR and HRR repair processes (Yu et al., 2022). According to studies, CTBP1-DT targets miR-3163 to increase ZNF217 expression, which in turn accelerates the progression of cervical cancer (Yang et al., 2020). According to Liu et al. high-grade serous ovarian cancer (HGSOC) tissues have a considerable overexpression of CTBP1-DT, which is associated with a poor prognosis of patients with HGSOC (Liu et al., 2021). Through competitive binding to miR-188-5p, it prevents MAP3K3 from being degraded, advancing HGSOC and serving a crucial regulatory function. A type of ferroptosis-related lncRNA called CTBP1-DT has recently been discovered, and it has the potential to treat head and neck squamous cell cancer (HNSCC) and forecast patients’ prognoses (Lu et al., 2022). In patients with prostate adenocarcinoma (PRAD), AC008610 was discovered to be an autophagy-related lncRNA that could predict disease-free survival (DFS) (Chen et al., 2022). However, AC034199.1, AC125437.1, AC107032.2, and AL024508.1 have not been reported in tumors so far. For the first time, we have confirmed the role of these 4 CRLs in ESCA, as far as we know. According to the calculated risk score, we divided the patients into the high risk group and the low risk group. Using the Kaplan-Meier curve to analyze the prognosis of each group. We discovered that the OS and PFS of the high-risk group were lower than those of the low-risk group. The survival time of ESCA patients decreased, and the number of fatalities rose as the risk score climbed. Then we carried out cox regression analysis and drew ROC curve, which showed that risk score was an independent risk factor for the prognosis of ESCA patients and the diagnostic effect was better than other clinical features, which also verified the reliability of our model. In addition, we have established a nomogram to more intuitively display the 1, 3, 5-year os for ESCA patients. Then we carried out GO and KEGG analysis, and the GO results showed that cuprotosis related lncRNAs was mainly related to antimicrobial humoral response, endopeptidase inhibitor activity and peptidase inhibitor activity, and KEGG pathway was mainly concentrated in cytokine-cytokine receptor interaction complement and coagulation cascades. The results showed that CRLs may play a role in ESCA through the above pathways. The TIDE score reflects the sensitivity of each risk group to immunotherapy. The effect of immunotherapy is poor in the low-risk group, and the clinical treatment of ESCA patients is of great significance.

Finally, we screened out the potential drugs and got 5 antineoplastic drugs: CP-466722, crizotinib, MS-275, KIN001-135, and WZ-1-84. CP-466722 is an effective, reversible ATM inhibitor, which can reduce the activity of purified ATM kinase to phosphorylate GST-p53 substrate. In addition, CP-466722 also shows inhibitory activity against abl and src kinase (Rainey et al., 2008). Crizotinib is an effective c-Met and ALK inhibitor and is considered the first-line drug for the treatment of advanced NSCLC with an ALK gene mutation (Jaboin et al., 2002; Shaw et al., 2020). MS-275 is a histone deacetylase inhibitor, which has a significant inhibitory effect on pediatric solid tumors *in vitro* and *in vivo* (Jaboin et al., 2002), but KIN001-135 and WZ-1-84 have not been reported. Despite the above results, our study still has some limitations and lacks more in-depth experiments *in vivo* and *in vitro*, which will carry out at a later stage. However, this study has been repeatedly analyzed and verified, and the results are accurate.

In summary, our study is the first to screen out CRLs to accurately predict the prognosis of patients with ESCA. These findings provide a new idea for tumor therapy and exploration of the mechanism of ESCA.

## Data Availability

The authors acknowledge that the data presented in this study must be deposited and made publicly available in an acceptable repository, prior to publication. Frontiers cannot accept an article title that does not adhere to our open data policies.
